# Phospho-dependent recruitment of NuA4 by MRX at DNA breaks regulates RPA dynamics during resection

**DOI:** 10.1186/1756-8935-6-S1-P93

**Published:** 2013-03-18

**Authors:** Olivier Jobin-Robitaille, Pierre Billon, Rémi Buisson, Valérie Côté, Hengyao Niu, Nicolas Lacoste, Patrick Sung, Steve Kron, Jean-Yves Masson, Jacques Côté

**Affiliations:** 1 Laval University Cancer Research Center, Hôtel-Dieu de Québec (CHUQ), 9 McMahon Street, Quebec City, Qc Canada G1R2J6; 2 Department of Molecular Biophysics & Biochemistry, Yale University School of Medicine, New Haven CT, USA; 3 Department of Molecular Genetics and Cell Biology, University of Chicago, Chicago, IL 60637, USA

## Background

The NuA4 histone acetyltransferase is a highly conserved multisubunit complex responsible for acetylation of nucleosomal histone H4 and H2A. Mutations in different NuA4 subunits create cell cycle arrest or delay in G2/M. The NuA4 complex is important for gene expression but also the efficient repair of DNA double strand breaks (DSBs) in vivo. NuA4 is rapidly recruited to chromatin surrounding a DSB in vivo at the same time histone H2A(X) is phosphorylated in the neighboring region (γ-H2AX). While we have shown that NuA4 can interact with phosphorylated H2A(X), we speculated that another interaction was required for the initial recruitment of NuA4 at the break. Based on the presence of an ATM-related factor within NuA4, the subunitTra1 (TRRAP), it was tempting to postulate that DSB sensing factors known to recruit ATM family PIKK factors could be responsible for NuA4 recruitment.

## Materials and methods

We performed chromatin IP experiments at an inducible DNA double-strand break (HO) in different mutant backgrounds. We used protein-protein (co-IP, pull down) and DNA-proteins (EMSA, pull downs) interactions assays to study the mechanistic and function of NuA4 activity in DNA double-strand break.

## Results

Mutation of Ku70/80 did not decrease NuA4 recruitment, but in fact even favored it specifically in G1. In contrast, deletion of Xrs2, Rad50 or Mre11, part of the MRX complex, strongly crippled NuA4 recruitment at DSBs. We found that Xrs2 FHA-BRCT domains directly interact with purified native NuA4 complex. Interestingly, this interaction is specifically detected when NuA4 is purified from cells treated with DNA damaging agents. A few subunits of NuA4 are phosphorylated in vivo in response to DNA damage, including Tra1, and interaction with Xrs2 is sensitive to phosphatase treatment. We analyzed how this targeting could be related to DNA end resection and homologous recombination. Expression of CDK inhibitor Sic1 greatly decreased NuA4 recruitment at DSBs and deletion of both Exo1 and Sgs1 had a striking effect, increasing NuA4 presence at the DNA break but stopping it from spreading to the usual ~4kb domain where NuA4 is normally detected. Thus, targeting of NuA4 does not require DNA end resection per se but needs it to spread to neighboring regions. Interestingly, RPA and NuA4 affect each other binding during DNA end resection and we show that NuA4 can displace RPA from single-strand DNA in vitro in an acetyl-CoA-dependent manner.

## Conclusion

These results indicate that NuA4 recruitment at DNA breaks occurs through a stepwise mechanism, initiated a phospho-dependent interaction with MRX, followed by spreading through DNA end resection where it regulates RPA dynamics.

